# The effect of composition, microfluidization and process parameters on formation of oleogels for ice cream applications

**DOI:** 10.1038/s41598-021-86233-y

**Published:** 2021-03-30

**Authors:** E. Silva-Avellaneda, K. Bauer-Estrada, R. E. Prieto-Correa, M. X. Quintanilla-Carvajal

**Affiliations:** Facultad de Ingeniería, Universidad de La Sabana, Km 7 vía autopista Norte, Bogotá, Colombia

**Keywords:** Oils, Chemical engineering

## Abstract

The use of oleogels is an innovative and economical option for the technological development of some food products, among them ice creams. The aim of this study was to establish the best processing conditions to obtain an emulsion which form oleogels with the lowest ζ-potential and average droplet size (ADS) for use as ice cream base. Using surface response methodology (SRM), the effects of three numerical factors (microfluidization pressure, oil and whey protein concentration, WP) and four categorical factors (oil type, temperature, surfactant, and type of WP) on formation of emulsions were assessed. The response variables were ζ, ADS, polydispersity index (PDI), viscosity (η), hardness, cohesiveness and springiness. Additionally, a numerical optimization was performed. Two ice creams containing milk cream and oleogel, respectively were compared under the optimization conditions. Results suggest oleogels obtained from the microfluidization of whey and high oleic palm oil are viable for the replacement of cream in the production of ice cream.

## Introduction

Among several fat-rich products, ice creams represent a very attractive product, consumed worldwide by all ages. However, the composition of the cream used to prepare ice creams is mainly saturated fats, called also solid fats, and its mainly purpose is to confer structure to the product^1^. Currently, consumers are increasingly aware of their dietary sources of fat, and vegetable oils such as coconut, soy and high oleic palm oil can be used as an alternative to solid fats by producing oil emulsions in water with a healthier fatty acid profile^2^. Iordache and Jelen^3^ concluded that microfluidized solutions of heated WPs resulted in the production of non-sedimented opaque suspensions, probably by the disintegration of insoluble aggregates into particles of substantially reduced size; when oil is added to these solutions and non-sedimented suspensions are formed, they can be called oleogels. These oleogels are defined as a high technological product produced by oil structuring food-approved polymers, that can be used to replace solid fat content and create optimal fat structure in ice creams^4,5^.

Whey protein (WP) has been recognized as a high value byproduct, and possess several advantages related to protein functionalities and the ability to form oleogels^6^. Proteins like β-lactoglobulin (~ 18.36 kDa) and α-lactalbumin (~ 14.17 kDa), present in the WP, display different functionalities such as emulsification and gelling according to the physicochemical treatment (pH, pressure, temperature) to which they are subjected^7^. The functionality of WP is closely related to the composition and amino acid sequence of these proteins, which allows a specific role or function in a food to be fulfilled, such as providing nutritional value or increasing solubility, gelation or emulsification in different matrices^8^.

One of the most widely used technologies for the production of emulsions is microfluidization (MF), which is a high energy method that uses high pressure to force a fluid through microchannels, thereby emulsifying it with the combined effects of cavitation, shear and impact^9^. In this work, microfluidization was chosen to emulsify WP with oils mentioned above and obtain oleogels, since it is a fully scalable process and has been implemented in different industries (food, pharmaceutical, cosmetic) for its high efficiency to decrease the particle size of different matrices^9,10^. Different studies suggest that MF affects the structure of the native protein and can produce significant differences when compared to denaturation and heat-induced aggregation, as MF can break intermolecular bonds, leading to protein fragmentation^11^. However, MF can greatly increase the hydrophobic nature of the surface of an absorbed protein layer by transforming aggregates of WPs into smaller aggregates, as has been demonstrated in emulsions. MF transforms these aggregates into smaller particles, leading to enhanced inter-droplet interactions and thereby increasing their potential application as emulsions in the preparation of gels^12^.

In recent years, oleogels have been of interest due to of their potential applications in food (as fat replacements), pharmaceuticals (as drug delivery systems) and cosmetics^13^. Solvent exchange routes^14^, temperature increases and solvent exchange^6^, and stirring at low temperature^13^ have been used to form oleogels, but the effects of MF on obtaining oleogels with different types of oils has not been studied. Finally, the ability to create and stabilize a dispersed food system depends mainly on its structural properties, extrinsic factors and process of creation. Therefore, the objective of this study was to evaluate through surface response methodology (SRM) the effect of various relevant industrial process parameters, such as temperature, pressure, concentration of surfactant, different oils and WP type, on the production of oleogels. These oleogels can be used as possible bases to produce ice cream and to thereby enhance their use through rheology and texture analysis.

## Materials and methods

### Materials

Three types of WP were collected from different regions of Colombia, WP1 supplied by Lácteos Castilac Ltda, WP2 supplied by Lactolife S.A.S from Cundinamarca, and WP3 supplied by Lácteos del campo Caqueteño S.A.S from Caquetá. Different oils were used for the preparation of emulsions: high oleic palm oil, HOPO (Fedepalma, Bogotá, Colombia); coconut oil, VCO (A of coconut, Colombia) and soy oil, SO (Carulla, Colombia). VCO and SO were acquired from a local market in Chia, Colombia.

### Spray drying

The collected whey was dried in a pilot scale spray dryer (GEA Process Engineering, Mobile Minor, GEA Niro, Denmark). During this process, an air inlet temperature of 215 °C and an air outlet temperature of 100 °C were controlled along with the whey inlet flow and the atomized air pressure (0.8–1 bar)^15^. A two-stream continuous-flow pneumatic nozzle (1 mm in diameter) with a capacity of 5 L h^−1^ was used as the atomizer system.

### Experimental design

To evaluate the emulsification and gelation functionality of WP, a type IV-optimal response surface design was used (Table 1) in the Design Expert Software V8.0.7.1 (Stat-Ease Inc., Minneapolis, MN, USA). Three numerical factors were evaluated at three levels: pressure (A) (65–130 MPa), oil concentration (B) (6–10% (w/w)), and WP concentration (C) (30–50% (w/w)); four categorical factors were evaluated: type of oil (D) (VCO, HOPO and SO)), temperature (E) (with and without T° control), surfactant (F) (with and without surfactant) and type of WP (G) (WP1, WP2 and WP3).

**Table 1:** Response optimization surface experimental design methodology for preparation of emulsions and adjusted variables to the model: ζ, ADS, PDI, *ƞ* for fresh and stored emulsions at 20 °C and 4 °C, and hardness, cohesiveness and springiness of oleogels.

Run	Numeric factors			Categoric factors				Response variables								
	A: Pressure (MPa)	B: Oil (%)	C: Whey (%)	D: Oil	E: Control T°	F: Lecithin	G: WP	ζ (mV)	ADS (nm)	PDI	ƞ_Fresh_ (mPa s)	ƞ_Stored 20 °C_ (mPa s)	ƞ_Stored 4 °C_ (mPa s)	Hardness (N)	Cohesiveness	Springiness
1	130.0	6.0	50.0	HOPO	No	Yes	WP2	− 22.8	377.5	0.47	315.6	573.6	721.4	147.3	1.51	0.44
2	65.0	6.0	33.1	SO	No	Yes	WP3	− 28.3	298.8	0.26	24.0	665.6	80.0	32.3	1.59	0.28
3	130.0	6.1	44.3	VCO	No	No	WP2	− 21.1	271.3	0.25	21.6	159.4	146.5	220.7	2.07	0.60
4	76.7	9.0	50.0	SO	No	Yes	WP1	− 29.3	274.6	0.26	188.7	655.8	427.5	61.0	1.40	0.03
5	89.6	7.1	31.3	SO	Yes	No	WP3	− 28.5	320.0	0.27	24.1	89.8	213.6	87.5	1.62	0.44
6	130.0	8.7	50.0	SO	Yes	No	WP1	− 26.7	290.6	0.39	581.4	2489.3	1547.4	168.7	1.43	0.44
7	65.0	8.5	50.0	VCO	No	No	WP2	− 17.7	237.8	0.19	47.4	318.5	210.0	242.8	1.95	0.54
8	120.3	6.0	30.0	VCO	Yes	No	WP1	− 26.7	252.8	0.23	35.8	864.1	292.2	46.8	1.90	0.12
9	65.0	10.0	30.0	HOPO	Yes	No	WP1	− 29.4	552.3	0.50	57.1	1676.8	553.6	40.7	1.52	0.01
10	65.0	9.9	39.2	SO	Yes	Yes	WP1	− 29.3	500.5	0.42	96.3	812.8	471.6	47.0	1.50	0.10
11	94.3	9.6	36.0	HOPO	No	Yes	WP3	− 28.7	344.2	0.32	45.8	397.0	341.1	116.2	1.62	0.32
12	124.2	7.0	39.0	VCO	No	Yes	WP3	− 26.4	217.3	0.16	53.8	358.7	97.9	75.8	1.47	0.48
13	130.0	6.0	30.0	HOPO	Yes	No	WP3	− 26.8	221.7	0.14	34.3	3416.3	345.2	63.7	1.52	0.61
14	104.0	6.0	30.0	SO	No	No	WP1	− 29.5	246.0	0.22	46.0	710.7	250.7	110.9	2.05	0.21
15	65.0	8.8	34.9	HOPO	Yes	Yes	WP2	− 19.6	379.0	0.30	6.1	50.3	7.16	51.4	1.49	0.01
16	65.0	10.0	47.4	SO	Yes	No	WP3	− 27.3	347.9	0.26	376.2	1334.0	1888.3	123.3	1.54	0.80
17	130.0	9.7	30.0	SO	No	Yes	WP3	− 28.3	338.9	0.32	36.7	494.1	211.2	39.4	1.43	0.06
18	114.9	7.1	32.1	SO	Yes	Yes	WP1	− 28.3	395.8	0.38	30.1	845.9	417.0	34.2	1.75	0.02
19	125.0	7.3	30.0	HOPO	Yes	No	WP2	− 27.2	215.3	0.12	6.3	9.2	10.5	116.8	1.54	0.51
20	87.4	6.0	50.0	HOPO	No	Yes	WP1	− 29.6	210.6	0.19	267.5	1910.9	922.9	88.0	1.32	0.16
21	128.6	7.4	42.0	SO	No	Yes	WP2	− 22.8	821.8	0.51	24.4	291.5	120.5	238.6	1.57	0.42
22	67.0	6.0	30.0	HOPO	No	Yes	WP1	− 28.7	291.7	0.23	47.1	5977.8	886.5	18.1	1.48	0.31
23	104.0	10.0	37.0	SO	Yes	Yes	WP2	− 19.8	521.0	0.48	6.7	13.1	7.90	103.5	1.66	0.04
24	81.9	6.0	41.0	VCO	No	Yes	WP2	− 17.3	422.7	0.44	9.3	15.5	9.90	76.4	1.77	0.11
25	130.0	8.0	30.4	HOPO	No	No	WP1	− 30.0	379.3	0.44	46.6	937.5	755.6	114.2	1.43	0.44
26	89.6	7.1	31.3	SO	Yes	No	WP3	− 27.5	283.7	0.25	77.4	81.8	346.2	83.1	1.43	0.60
27	94.3	9.6	36.0	HOPO	No	Yes	WP3	− 29.2	319.7	0.26	52.0	551.8	242.6	77.9	1.63	0.19
28	98.2	7.2	37.5	HOPO	No	No	WP3	− 27.9	236.1	0.15	90.0	1483.8	570.1	102.5	1.45	0.81
29	123.5	6.0	48.9	SO	Yes	Yes	WP3	− 28.5	193.2	0.12	92.4	716.4	202.9	115.0	1.40	0.76
30	130.0	10.0	30.0	VCO	No	Yes	WP2	− 20.2	787.8	0.46	5.7	23.9	6.7	71.9	1.67	0.09
31	101.4	10.0	50.0	VCO	No	No	WP1	− 29.3	310.2	0.36	603.0	4730.6	2401.6	51.1	1.63	0.32
32	83.6	9.5	31.5	VCO	No	Yes	WP1	− 27.7	431.0	0.46	97.9	774.2	315.9	57.1	1.52	0.11
33	130.0	7.7	49.3	VCO	Yes	Yes	WP2	− 16.4	700.3	0.52	269.7	1451.7	1852.3	225.9	1.60	0.30
34	71.5	7.4	50.0	HOPO	Yes	Yes	WP3	− 27.5	279.1	0.29	94.5	1310.3	1171.1	116.1	1.47	0.74
35	124.2	7.0	39.0	VCO	No	Yes	WP3	− 27.2	228.5	0.16	94.5	883.2	188.1	78.1	1.48	0.42
36	65.0	10.0	50.0	HOPO	No	No	WP1	− 30.5	296.6	0.22	562.3	2295.6	1622.9	57.8	2.15	0.46
37	65.7	7.8	47.7	VCO	Yes	Yes	WP1	− 28.7	535.3	0.51	221.0	571.8	527.5	56.4	1.40	0.08
38	94.3	10.0	50.0	HOPO	No	No	WP2	− 22.0	228.6	0.14	108.6	323.7	187.7	280.5	1.51	0.33
39	72.1	9.2	36.4	VCO	Yes	No	WP2	− 21.8	320.1	0.24	8.1	16.5	8.84	78.3	1.58	0.18
40	100.8	6.3	42.6	HOPO	Yes	No	WP1	− 28.2	203.5	0.14	184.2	912.8	669.9	88.3	1.47	0.58
41	114.6	7.9	40.4	VCO	No	No	WP1	− 28.4	304.8	0.28	201.3	961.9	328.4	179.5	1.44	0.38
42	130.0	10.0	45.9	HOPO	Yes	Yes	WP1	− 28.4	299.1	0.27	167.3	705.7	362.2	75.3	1.40	0.10
43	100.8	6.3	42.6	HOPO	Yes	No	WP1	− 28.5	241.9	0.22	133.0	670.9	317.4	66.8	1.42	0.56
44	114.9	7.1	32.1	SO	Yes	Yes	WP1	− 27.8	283.3	0.24	45.3	748.6	196.4	39.9	1.82	0.04
45	65.0	6.8	41.9	VCO	No	No	WP1	− 29.2	282.0	0.25	188.3	1021.7	374.6	111.9	1.41	0.49
46	130.0	6.0	50.0	VCO	No	Yes	WP1	− 23.8	172.3	0.18	299.9	2452.8	1837.5	73.9	1.51	0.03
47	130.0	10.0	40.0	SO	No	No	WP1	− 30.5	346.3	0.42	208.2	1021.8	542.8	122.1	1.21	0.37
48	65.0	6.0	34.9	HOPO	No	No	WP2	− 24.9	236.8	0.16	6.00	6.55	6.39	60.8	1.55	0.06
49	130.0	10.0	50.0	HOPO	No	No	WP3	− 27.7	226.6	0.18	410.3	3177.7	634.2	163.6	1.73	0.57
50	89.7	6.7	50.0	SO	No	No	WP3	− 26.5	212.8	0.15	347.4	1479.4	2385.5	159.3	1.79	0.69
51	65.0	9.2	30.7	SO	No	No	WP2	− 28.1	295.3	0.15	5.80	7.90	6.21	93.1	1.61	0.46
52	80.9	6.0	50.0	VCO	Yes	No	WP3	− 27.8	225.4	0.19	110.1	2449.6	790.7	155.0	1.54	0.60
53	130.0	9.8	38.0	VCO	Yes	No	WP3	− 26.7	348.5	0.37	120.2	551.1	278.1	161.4	1.64	0.28
54	65.0	10.0	50.0	VCO	No	Yes	WP3	− 28.3	288.6	0.34	152.8	1339.0	390.0	80.7	1.45	0.71
55	65.0	6.0	49.0	SO	Yes	No	WP2	− 18.8	364.8	0.35	53.9	369.5	216.9	237.6	1.91	0.53
56	81.3	8.6	30.0	VCO	Yes	Yes	WP3	− 27.7	362.1	0.34	47.7	554.6	188.1	99.7	1.88	0.26
57	81.3	9.6	30.0	VCO	No	No	WP3	− 25.7	393.6	0.41	157.3	2793.9	259.6	49.4	1.55	0.31

The response variables were ζ (mV), ADS (nm), PDI, and η_Fresh_ (mPa s), η_Stored 20 °C_ (mPa s), η_Stored 4 °C_ (mPa s), hardness (N), cohesiveness (N mm) and springiness (mm). A statistical significance test was used in the total error criteria with a confidence level of 95%. Significant factors in the model were identified through analysis of variance (ANOVA) with a confidence level of 95%. Model fit was evaluated with the R^2^ value and the adjusted R^2^ value. The graphic and numerical optimization of the Design Expert software was used for response optimization. One optimization system was formulated for an emulsion with the lowest ζ and ADS for use as an ice cream base.

### Preparation of the emulsions

In accordance with the experimental design (Table 1), oil with or without surfactant (soy lecithin) was slowly added to the WP solution at a concentration of 1% (w/w) with respect to the oil; the samples were homogenized in an Ultra-Turrax (T-25 basic, IKA, Staufen, Germany) homogenizer with a S25 KV-25G geometry at 15,000 rpm for 10 min. A volume of 180 mL of each run was microfluidized at the pressure indicated in the experimental design (65–130 MPa) for 3 cycles with temperature controlled at 5 °C or uncontrolled in a Microfluidizer (Microfluidics, Newton, USA)^3^. Uncontrolled temperatures ranges between 5 and 42 °C. Approximately 40 mL of each fresh emulsion were placed in 50-mL Falcon tubes and stored during 3 days at room temperature (20 °C) and in a refrigerator (4 °C) for further analysis.

### ADS, PDI and ξ

The ADS and PDI of the fresh emulsions were obtained by dynamic light scattering (DLS) dispersion using a disposable cell (DTS0012). The net electric charge of the WPC solutions was determined by measuring the ξ using a foldable capillary cell (DTS1070). Measurements were performed on a Zetasizer Nano ZS (Malvern Instruments, Malvern, UK). For the measurements, 1: 1000 dispersions were prepared in accordance with the methodology of Qian et al*.*^16^*,* and the samples were equilibrated in the equipment at 25 °C. Each measurement was performed in triplicate with a scattering angle of 173°.

### Determination of viscosity

The viscosity of fresh emulsions and those stored at 20 °C and 4 °C was measured on a rheometer (Para-Physica MCR 502, Anton Paar GmbH, Ostfildern, Germany), and a profiled plate geometry (PP50) with a gap of 0.5 mm and a speed between 1 and 100 s^−1^ was performed. Measurements were carried out at room temperature. The viscosity was reported at a shear rate of 25 s^−1^.

### Preparation of oleogels

Approximately 15 g of fresh emulsions obtained from each run were placed in stainless steel molds to heat-cure in a water bath at 90 °C for 90 min and then placed in an ice bath for 30 min in order to obtain the oleogels^17^.

### Analysis of texture parameters

The WP oleogels resulting from each run were compressed axially in two consecutive cycles at a test speed of 2 mms^-1^, with a flat geometry 25 mm in diameter. Measurements were performed in triplicate. A texture profile analysis (TPA) was carried out to determine the hardness, cohesiveness and springiness of the gels as proposed by Friedman and collaborators^18^. Only significant variables were reported. Assays were performed at room temperature with a TA.XT plus texture analyzer (Stable Micro Systems, Godalming, UK).

### Ice cream formulation and analysis

Two different ice cream formulations were produced. The fat used in the first ice cream was milk cream (Parmalat Colombia Ltda, Bogotá, Colombia), and the second used the emulsion with the best conditions as determined by numerical optimization (lowest ζ and ADS). In an Ultra-Turrax (T-25 basic, IKA) at 8000 rpm, 240 mL of whole milk, 174.6 g of sugar and 0.20 g of salt were homogenized; separately, the respective fat and vanilla essence were homogenized under the same procedure, after which all ingredients were mixed (Imusa, Bogotá, Colombia) and allowed to cool (5 °C) for 12 h. The mixture was then added to an Ice Cream Maker Model ICE-21R (Cuisinart, New Jersey, USA) and stirred for 20 min to form ice creams. Once soft ice creams were formed, they were placed in a container and frozen at − 17 °C for 48 h. The ice creams were subjected to a texture profile analysis using a TA.XT plus texture analyzer (Stable Micro Systems, Godalming, UK) in order to determine hardness, cohesiveness, springiness, adhesiveness, chewiness and gumminess. Furthermore, the viscosity of the ice creams was measured using the same conditions described for the determination of viscosity of the oleogels.

## Results and discussion

### Effects of MF on the ζ, ADS, PDI, η of fresh emulsions

#### ζ-potential of fresh emulsions

The influence of various factors on the ζ of fresh emulsions was determined. Values of ζ ranged from − 16.4 to − 30.5 mV (Table 1). The factors that significantly affected ζ were oil concentration (B), WP concentration (C), type of oil (D), temperature (E), surfactant (F), type of WP (G), WP concentration squared (C^2^), and the interactions AD, AG, BE, BG, CD, CG, DE, DF, DG, EG and FG. These values were adjusted to a quadratic model with an R^2^ of 0.99 (P < 0.05), and the lack of fit was not significant (Table 2). According to the results obtained, the presence of soy lecithin (F) is significant considering the ζ value of fresh emulsions. This agrees with the results of Wang et al.^19^, who found that increasing the concentration of lecithin in an emulsion with whey protein isolate (WPI) and peony oil resulted in a ζ that was approximately 10% more negative than that of an emulsion without lecithin. This can be attributed to the fact that the conformational changes on WP induced by lecithin binding could promote the exposure of negatively charged WP residues and therefore decrease ζ.

**Table 2:** ANOVA for the adjusted variables to response optimization design: ζ, ADS, PDI, and *ƞ* for fresh emulsions.

Independent factor	ζ*-*Potential (mV)			ADS (nm)			PDI			η_Fresh_ (mPa s)		
	SS	df	*P*-value	SS	df	*P*-value	SS	df	*p*-value	SS	df	*P*-value
Model	734.19	46	**< 0.0001**	9.9 × 10^5^	46	**0.002**	0.749	46	**0.0035**	1.2 × 10^6^	46	**< 0.0001**
A (Pressure)	0.01	1	0.86	2.5 × 10^3^	1	0.42	0.003	1	0.38	1.2 × 10^4^	1	**0.001**
B (Oil %)	2.13	1	**0.04**	7.9 × 10^4^	1	**0.0008**	0.084	1	**0.0004**	1.0 × 10^4^	1	**0.014**
C (Whey %)	12.95	1	**0.0001**	3.5 × 10^4^	1	0.01	0.021	1	**0.026**	3.2 × 10^5^	1	**< 0.0001**
D (Type of oil)	37.90	2	**< 0.0001**	2.6 × 10^4^	2	0.06	0.038	2	**0.02**	2.8 × 10^2^	2	0.8867
E (Control T°)	4.56	1	**0.005**	8.9 × 10^3^	1	0.14	0.013	1	0.06	4.3 × 10^1^	1	0.9849
F (Lecithin)	5.07	1	**0.004**	3.7 × 10^4^	1	**0.009**	0.026	1	**0.015**	6.5 × 10^4^	1	**< 0.0001**
G (WP)	411.80	2	**< 0.0001**	1.4 × 10^5^	2	**0.0004**	0.084	2	**0.0013**	1.5 × 10^5^	2	**< 0.0001**
AB	0.28	1	0.40	8.0 × 10^3^	1	0.16	0.009	1	0.12	1.7 × 10^4^	1	**0.0031**
AD	8.50	2	**0.002**	8.4 × 10^3^	2	0.34	0.005	2	0.46	9.4 × 10^2^	2	0.68
AG	18.27	2	**0.0001**	6.8 × 10^4^	2	**0.005**	0.019	2	0.09	2.1 × 10^3^	2	0.43
BC	0.05	1	0.72	3.0 × 10^2^	1	0.78	0.016	1	**0.04**	5.1 × 10^3^	1	0.061
BD	1.17	2	0.25	2.7 × 10^4^	2	0.06	0.010	2	0.24	2.1 × 10^4^	2	**0.005**
BE	2.29	1	**0.03**	9.4 × 10^2^	1	0.61	0.003	1	0.37	4.1 × 10^3^	1	0.09
BF	0.57	1	0.24	4.6 × 10^2^	1	0.72	0.003	1	0.32	1.9 × 10^4^	1	**0.002**
BG	5.60	2	**0.009**	1.9 × 10^3^	2	0.77	0.036	2	**0.02**	3.3 × 10^4^	2	**0.001**
CD	5.01	2	**0.01**	1.2 × 10^4^	2	0.24	0.003	2	0.58	3.8 × 10^3^	2	0.242
CF	0.71	1	0.19	6.2 × 10^3^	1	0.21	0.009	1	0.11	5.1 × 10^4^	1	**< 0.0001**
CG	41.03	2	**< 0.0001**	1.0 × 10^4^	2	0.27	0.039	2	**0.02**	6.4 × 10^4^	2	**< 0.0001**
DE	12.77	2	**0.0005**	1.1 × 10^4^	2	0.26	0.011	2	0.22	1.1 × 10^4^	2	**0.038**
DF	4.29	2	**0.02**	7.1 × 10^3^	2	0.4	0.002	2	0.69	1.5 × 10^4^	2	**0.02**
DG	12.74	4	**0.003**	3.6 × 10^4^	4	0.10	0.018	4	0.29	1.5 × 10^4^	4	0.06
EG	5.32	2	**0.01**	2.4 × 10^4^	2	0.075	0.001	1	0.67	2.6 × 10^4^	2	**0.003**
FG	31.07	2	**< 0.0001**	9.7 × 10^4^	2	**0.001**	0.009	2	**0.001**	5.6 × 10^4^	2	**0.0001**
C^2^	2.85	1	**0.02**	1.7 × 10^3^	1	0.50	0.002	1	0.45	4.0 × 10^4^	1	**0.0002**
Residual	3.63	10	–	3.5 × 10^4^	10	–	0.030	10	–	1.2 × 10^4^	10	–
Lack of fit	2.62	5	0.16	2.7 × 10^4^	5	0.11	0.015	5	0.49	7.8 × 10^3^	5	0.21
Pure Error	1.00	5	–	8.1 × 10^3^	5	–	0.015	5	–	3.7 × 10^3^	5	–
R^2^	0.99	–	–	0.97	–	–	0.96	–	–	0.99	–	–
Adj R^2^	0.97	–	–	0.80	–	–	0.78	–	–	0.94	–	–

Values in bold represent *p* values lower than 0.05 which have a significant effect

Another factor that affected the stability of emulsions was the oil concentration, as lower values of ζ were obtained at higher oil concentrations (Table 1). This behavior may occur because the oils used are composed of medium chain (VCO) and long chain triglycerides (HOPO and SO) with fatty acids of 10 to 18 carbon atoms in length and have low molecular weights^20^. Because VCO, HOPO and SO oils have proportions of monounsaturated (oleic) and polyunsaturated fatty acids (linoleic and linolenic), their structure (double bonds) makes them more sensitive to extrinsic factors such as increased temperature and pressure, which also catalyzes protein hydrolysis.

The interactions between the type of whey and all the factors evaluated in the experimental design were significant. This was verified experimentally, as in emulsions made with WP2 whey, although no separation of phases was observed during two weeks of evaluation, oleogels were never formed and these emulsions exhibited the highest values of ζ, in contrast to WP1 and WP3 whey samples, which always formed oleogels. This may be because the steric repulsion between dispersed oil droplets is not high enough to overcome the intermolecular attraction force between the droplets and the energy barrier. As a result, the emulsion gels after a few days because the WPs tend to aggregate together with the oil droplets as the temperature increases during MF^21^.

#### ADS and PDI of fresh emulsions

The results obtained from ADS and PDI are shown in Table 1. A small ADS (nm scale) is of interest because droplet size is strongly correlated with high stability in conventional emulsion systems^22^. The factors that were significant in the ADS were oil concentration (B), surfactant (F), type of WP (G) and pressure-type of WP (AG) and surfactant-type of WP (FG) interactions, and the values obtained were fitted to a quadratic model with an R^2^ of 0.97 (*P* < 0.05), in which the lack of fit was not significant (Table 2). The results demonstrate that ADS, and the charge of oil droplets in emulsions can be controlled by selecting an appropriate emulsifier (type and concentration) and an appropriate homogenization procedure (MF, cycles, temperature)^23^.

**Figure 1:**
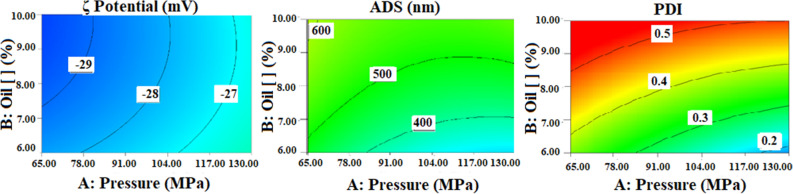
Graphs of the models of *ζ*, ADS and PDI in function of pressure (x axis) and oil concentration (y axis).

The ADS of the emulsions was significantly reduced by the pressure used in the microfluidizer (Fig. 1). The same results were obtained by Dissanayake et al.^24^, who concluded that MF directly reduces the particle size in emulsions made with WP powder. High shear homogenizers such as a Microfluidizer promote two processes: droplet break-up and re-coalescence, which when combined are enough to decrease ADS, depending on the oil concentration, presence of surfactant and WP type. Similarly, fat globules are intimately connected to proteins, as highly interconnected and dense networks were formed in most emulsions^25^. On the other hand, in most emulsions made with WP2 whey, oleogels were not formed, probably due to the large particle size of these emulsions, which led to a lower capacity to form gels^6^.

PDI is a dimensionless parameter describing the relative width or variance of the particle size distribution^17^. The PDI can assume values lower than 0.05 for monodisperse distributions and values above 0.7 in samples with very large size distributions^9^. As seen in Table 1, the PDI values varied between 0.12 and 0.52, which indicate that the emulsions were polydisperse^26^. However, a relatively narrow droplet size distribution was observed, which suggests that the MF method used to obtain the emulsions was satisfactory^9^. The oil concentration (B), the WP concentration (C), the type of oil (D), the surfactant (F), and the type of WP (G) significantly affected the PDI values (Table 2). This is because the proteins present in the WP are emulsifiers, as they are amphiphilic (having hydrophobic and hydrophilic residues) and have active surfaces, enabling them to form strong and cohesive membranes around the droplets, thereby stabilizing them in emulsions^26^. The PDI values were adjusted to a quadratic model with an R^2^ of 0.96 (*P* < 0.05), and the lack of fit was not significant (Table 2). Moreover, the interactions BC, BG, CG and FG significantly influenced PDI values.

#### η of fresh emulsions

The rheological behavior of dairy products is complex and depends on the temperature, concentration and physical state of the dispersed phase^7^. The η behavior of proteins in emulsions is the result of several factors, including the size, shape and polydispersity of protein molecules and their aggregates, protein-solvent interactions, hydrodynamic volume and the molecular flexibility of proteins in their hydrated state^27^. According to the results for fresh emulsions (Table 1), factors that significantly influenced the η of the fresh emulsions were pressure (A), oil concentration (B), WP concentration (C), surfactant (F), type of WP (G), WP concentration squared (C^2^) and the interactions AB, BD, BF, BG, CF, CG, DE, DF, EG and FG (Table 2). The values were adjusted to a quadratic model with an R^2^ of 0.99 (*P* < 0.05), and the lack of fit was not significant (Table 2). The concentration of WP was significant, as above the critical concentration, the distances between particles are smaller and colloidal interactions are stronger, which leads to additional associations of these aggregates and, eventually, a higher η^28^.

#### Effects of MF on the η of oleogels stored at different temperatures

Many food products can be classified as gelled emulsions or emulsion gels. This applies to protein-based oil-in-water emulsions, which can be converted into soft-solids by food processing operations such as heating, acidification, pressure, and enzymatic action. However, the denaturation and aggregation of globular proteins by high-pressure treatments, such as MF, provides an alternative to thermal treatment for the gelation of protein-based colloidal systems^27^.

**Figure 2:**
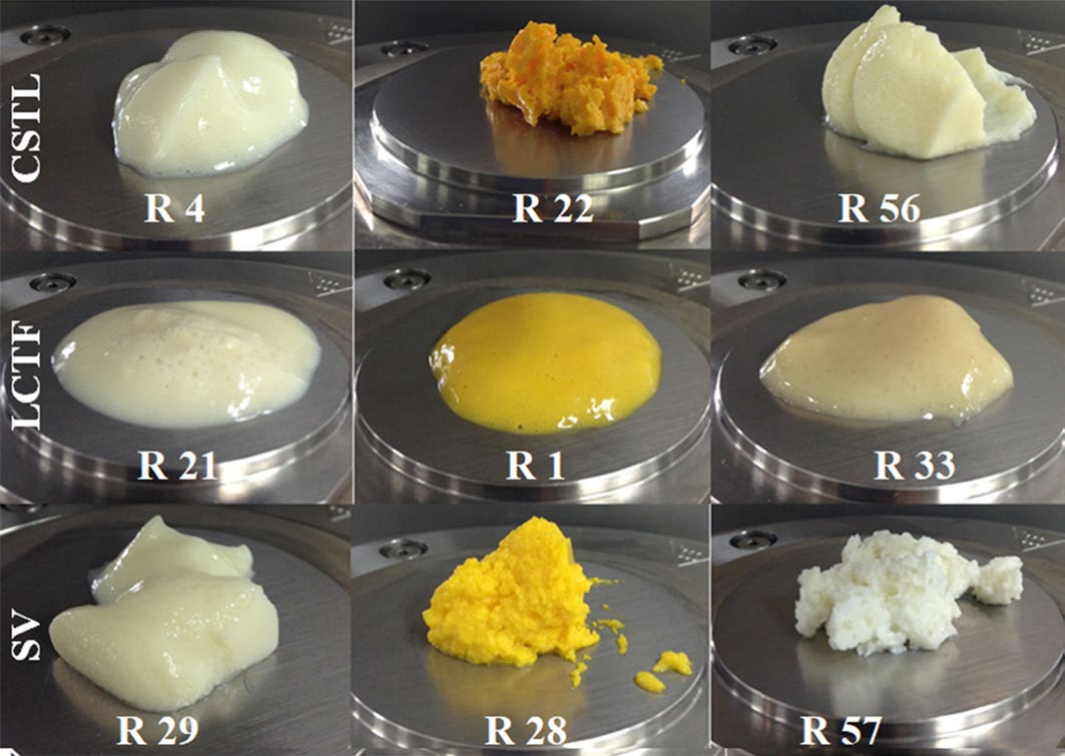
Oleogels formed with different WP and oils by MF. Rows: 1. WP1, 2. WP2 and 3.WP3, columns: 1. SO, 2. HOPO, 3. VCO. R corresponds to the run (emulsion) of the experimental design.

Of the resulting emulsions, 63% were highly stable with no phase separation, as MF treatment resulted in more structured systems^12^. Likewise, MF generates more homogeneously sized particles, which prevented emulsions from separating^3^; even emulsions that gelled without further heat treatment were obtained (Fig. 2).

Similar to Zhu et al.^29^, emulsions that were stored at room temperature formed oleogels due to the flocculation of oil droplets, which led to the formation of a gel-type network (oleogel) in these emulsions. The oil concentration and its interaction with the other evaluated parameters were the main factors that most greatly influenced the viscosity variable (Table 3). As mentioned above, this can be attributed to the effect of temperature and pressure on the double bonds of the mono and polyunsaturated components of VCO, HOPO and SO oils.

**Table 3:** ANOVA for the adjusted variables to response optimization design η for stored emulsions at 20 °C and 4 °C, and hardness, cohesiveness and springiness of oleogels.

Independent factor	η_stored 20°C_ (mPa s)			η_stored 4°C_ (mPa s)			Hardness (N)			Cohesiveness			Springiness		
	SS	df	*p*-value	SS	df	*p*-value	SS	df	*p*-value	SS	df	*p*-value	SS	df	*p*-value
Model	7.5 × 10^7^	46	**0.0015**	1.8 × 10^7^	46	**0.045**	2.0 × 10^5^	46	**0.006**	1.95	46	**0.023**	2.937	46	**0.003**
A	8.7 × 10^4^	1	0.57	1.3 × 10^5^	1	0.37	1.4 × 10^4^	1	**0.003**	0.009	1	0.42	0.012	1	0.33
B	7.2 × 10^5^	1	0.12	1.7 × 10^4^	1	0.74	9.3 × 10^2^	1	0.33	0.09	1	0.024	0.111	1	**0.012**
C	1.1 × 10^5^	1	0.52	3.5 × 10^6^	1	**0.0006**	4.5 × 10^4^	1	**< 0.0001**	0.03	1	0.19	0.307	1	**0.0005**
D	4.2 × 10^6^	2	**0.007**	7.1 × 10^4^	2	0.79	2.7 × 10^3^	2	0.27	0.10	2	0.05	0.101	2	0.045
F	6.1 × 10^5^	1	0.15	4.0 × 10^5^	1	0.13	2.0 × 10^4^	1	**0.0008**	0.05	1	0.07	0.455	1	**< 0.0001**
G	1.1 × 10^7^	2	**0.0002**	2.2 × 10^6^	2	**0.0099**	3.9 × 10^4^	2	**0.0002**	0.13	2	**0.03**	0.715	2	**< 0.0001**
AC	3.0 × 10^6^	1	**0.006**	2.4 × 10^4^	1	0.69	1.9	1	0.97	0.06	1	0.0516	0.002	1	0.73
BC	5.0 × 10^6^	1	**0.001**	5.1 × 10^3^	1	0.86	2.2 × 10^3^	1	0.15	0.007	1	0.4709	0.009	1	0.40
BD	4.2 × 10^6^	2	**0.007**	9.6 × 10^5^	2	0.08	5.5 × 10^3^	2	0.09	0.19	2	0.0101	0.067	2	0.10
BF	2.8 × 10^6^	1	**0.007**	3.9 × 10^5^	1	0.14	1.0 × 10^3^	1	0.31	0.01	1	0.3811	0.002	1	0.68
BG	2.3 × 10^5^	2	0.64	4.2 × 10^5^	2	0.29	4.8 × 10^3^	2	0.12	0.20	2	0.0092	0.011	2	0.64
CD	3.5 × 10^6^	2	**0.012**	5.8 × 10^5^	2	0.19	5.7 × 10^2^	2	0.73	0.05	2	0.1846	0.003	2	0.88
CF	2.5 × 10^6^	1	**0.010**	1.0 × 10^6^	1	**0.02**	4.8 × 10^2^	1	0.48	0.29	1	0.0007	0.020	1	0.22
CG	2.4 × 10^5^	2	0.63	8.8 × 10^4^	2	0.75	2.3 × 10^4^	2	**0.002**	0.02	2	0.5316	0.081	2	0.07
DF	2.2 × 10^6^	2	**0.042**	4.4 × 10^5^	2	0.27	7.5 × 10^2^	2	0.67	0.002	2	0.9265	0.056	2	0.14
DG	5.5 × 10^6^	4	**0.013**	9.9 × 10^5^	4	0.23	4.5 × 10^3^	4	0.36	0.21	4	0.0311	0.100	4	0.15
EG	2.0 × 10^6^	2	0.05	4.2 × 10^5^	2	0.29	7.9 × 10^3^	2	**0.04**	0.0001	2	0.9942	0.020	2	0.46
FG	2.7 × 10^6^	2	**0.03**	5.2 × 10^5^	2	0.22	2.9 × 10^3^	2	0.25	0.01	2	0.5807	0.034	2	0.28
B^2^	2.4 × 10^6^	1	**0.01**	6.5 × 10^3^	1	0.83	6.5 × 10^3^	1	**0.02**	0.02	1	0.1940	0.055	1	0.06
C^2^	8.5 × 10^6^	1	**0.0002**	1.2 × 10^6^	1	**0.02**	9.7 × 10^2^	1	0.32	0.11	1	0.0149	0.053	1	0.06
Residual	2.5 × 10^6^	10	–	1.5 × 10^6^	10	–	9.0 × 10^3^	10	–	0.13	10	–	0.118	10	–
Lack of fit	2.3 × 10^6^	5	**0.007**	1.4 × 10^6^	5	**0.007**	8.0 × 10^3^	5	**0.019**	0.11	5	0.05	0.095	5	0.07
Pure Error	1.8 × 10^5^	5	–	1.0 × 10^5^	5	–	9.9 × 10^2^	5	–	0.02	5	–	0.023	5	–
R^2^	0.97	–	–	0.93	–	–	0.96	–	–	0.94	–	–	0.96	–	–
Adj R^2^	0.82	–	–	0.58	–	–	0.76	–	–	0.66	–	–	0.78	–	–

Values in bold represent *p* values lower than 0.05 which have a significant effect

The stability of emulsions stabilized with WP proteins^30^ depends on the ADS, PDI, type of oil and crystallization of the fats present. Also, when emulsions are cooled to temperatures where part of the fat phase becomes crystalline, fat crystals from one droplet may penetrate into another droplet leading to emulsion destabilization by partial coalescence^31^. During our experiments, we observed that some of the emulsions containing VCO had crystallized particles, and as found by McClements^32^, changes in the physicochemical properties (rheology and appearance) of these oleogels were observed. The changes in appearance observed were the formation of small crystals in the oleogels and changes in color, which may have been the result of Maillard reactions catalyzed by the denaturation of the WPs^33^, a side effect of MF.

Changes in the η when emulsions were stored at different temperatures (Table 1) could occur due to the shape and size of the crystals present, as, according to the results from emulsions stored at room temperature (20ºC), the factors that significantly influenced the η were type of oil (D), type of WP (G), oil concentration squared (B^2^), WP concentration squared (C^2^) and the interactions between AC, BC, BD, BF, CD, CF, DF, DG and FG (Table 3). Significant factors affecting the η of emulsions stored at 4 °C were WP concentration (C), type of WP (G), WP concentration squared (C^2^) and the interactions between CF. The values obtained were adjusted to a quadratic model with R^2^ values of 0.96 and 0.92 (*P* < 0.05), respectively. As MF is a high pressure process, it causes temperature increases, producing the thermal denaturation of β-Lg, which is characterized by the alteration of secondary and tertiary structures, thereby exposing hydrophobic residues to solvent, which leads to the aggregation and intermolecular bonding of the matrix components^34^ and could be observed in the formation of oleogels in the majority of emulsions.

Most of the emulsions stored at 4 °C had lower values of η than emulsions that were stored at room temperature (Table 1), possibly *because* in low temperature emulsions the speed and frequency of collisions between droplets are lower, which reduces the growth of droplet size in the emulsion^9,35^ and the aggregation rate of WPs. When used as emulsions, WP aggregates influence various factors, including surface hydrophobicity, protein flexibility, electrostatic interactions, steric effects, ionic strength and protein concentration^36^. For all of the above reasons, MF greatly improves the efficiency of emulsification and gelling through a combined mechanism of cavitation, shear and impact and therefore has great potential for the preparation of oleogels^12^.

### Effects of heat treatment on different texture parameters of emulsions

The effect of the heat treatment (90 °C) of emulsions on texture parameters such as hardness, cohesiveness and springiness was evaluated (Table 1). The factors that significantly influenced hardness were pressure (A), WP concentration (C), surfactant (F), type of WP (G), oil concentration squared (B^2^) and CG and EG interactions (Table 3), which define the intensity of the forces present in the gel (hydrophobic, hydrophilic and covalent bonds) and compromise the structure of proteins in the WP^37^. The hardness values were adjusted to a quadratic model with a R^2^ of 0.96 (*P* < 0.05), and the lack of fit was significant. The experimental results agree with the results obtained by Devi et al.^38^, who concluded that the factors that affect hardness in gels made with WP are the composition of WP, pressure, concentration of WP, time of pressure treatment, temperature and pH.

The factors that significantly affected cohesiveness were oil concentration (B), type of WP (G), WP concentration squared (C^2^) and the interactions of AB, BD, BG and CF (Table 3). These factors were decisive and responsible for promoting intermolecular polymerization through the formation and increase of cohesiveness within gels^39^. The experimental values were adjusted to a quadratic model with an R^2^ of 0.94 (*P* < 0.05), and the lack of fit was not significant.

The significant factors affecting springiness were similar to those for hardness: oil concentration (B), WP concentration (C), type of oil (D), surfactant (F), G (type of WP) and AG interaction (Table 3). This may be because the gels made with denatured WP had greater resistance due to the presence of intermolecular di-sulfide bonds, which are believed to confer springiness to WP gels^40^. This result can also be attributed to a physical phenomenon resulting from the increased chain length of the protein aggregates after heat treatment, which restricts mobility by cross-linking and therefore increases non-covalent interactions^40^. The obtained values were adjusted to a quadratic model with an R^2^ of 0.94 (*P* < 0.05), and the lack of fit was not significant.

### Oleogel optimization and further elaboration of ice cream

After experiments were performed and to obtain an emulsion with the lowest ζ and ADS for use as an ice cream base, numerical and graphical optimization of the Design Expert software was used for response optimization following the desirability and criterion^41^. Numerical optimization yielded 100 different solutions (data not shown) with a desirability between 0.953 and 1. Solution 65 was chosen, which had the following parameters: pressure 120.97 MPa; oil concentration 6.80%; concentration of WP 46.03%; oil type HOPO; no temperature control during MF; surfactant and WP1; a desirability of 0.99′ and prediction ζ and ADS values of − 30.19 mV and 172.25 nm, respectively. The experimental errors for ζ and ADS were 3.2% and 0.6%, respectively. This solution was chosen due to the ratio oil:WP and the lack of control temperature needed during the MF that simplifies the process allowing the production of an oleogel that exhibits the desired characteristics.

High oleic palm oil (HOPO) is a hybrid between *Elaeis oleifera* and *Elaeis guineensis,* and its oleic acid content is significantly different from African palm oil (54.6 ± 1.0 vs. 41.4 ± 0.3)^40^. HOPO is more unsaturated and has nutritional and technical advantages^42^. African palm oil, mainly extracted from *E. guineensis*, has been a source of criticism in recent years, as it is a rich source of saturated fatty acids (SFA), specifically palmitic acid, and its use in food products has been somewhat discouraged. The results of Mozzon et al.^43^, demonstrate that the partial dietary replacement of saturated palmitic acid with monounsaturated oleic acid can be achieved by consuming HOPO instead of African palm oil, without the need for any fractionation processes to separate olein and stearin fractions. On the other hand, presence of these type of oil in oleogels can change the functional properties of proteins, which introduces the possibility of improving the rheological and sensory properties (texture) of food products such as ice cream^34^.

Rheological information helps to predict the mouthfeel, storage stability and durability, also to verify consistency, perform quality control and accelerate product development, for example, in dairy products. All of these attributes can be complemented with a TPA, which aims to simulate the repeated biting or chewing of a food in the mouth^44^ and provides parameters of hardness, springiness, adhesiveness, cohesiveness, chewiness and gumminess. Thus, as HOPO has excellent nutritional properties, it was used for the development of an ice cream prototype, besides being the oil, in which according to the optimization, it produced the emulsion with the lowest ζ and ADS. To determine whether the best emulsion resulting from optimization could replace the fat (cream) used in ice cream preparation, two different ice creams were made, one with milk cream (traditional ice cream) and one with the optimized emulsion. These ice creams were characterized according to their textural and rheological properties.

Ice cream’s resistance to the mechanical forces imparted by the tongue, the upper palate and the teeth, and the general perception of the texture of ice cream are part of its sensory acceptance. TPA can quantify the factors that are part of this acceptance^45^. The results obtained from TPA for the two ice creams (traditional and optimized) studied, were analyzed by ANOVA (Table 4). Trasditional ice cream presented lower cohesiveness, chewiness and gumminess and higher adhesiveness compared to optimized ice cream (P < 0.05). On the other hand, no significant differences (P > 0.05) were obtained between hardness and springiness of the two ice creams studied. Results suggests that the oleogels obtained from the microfluidization of whey and HOPO are a viable option for the replacement of raw materials (cream) in the production of ice cream, with added value provided by WP and HOPO.

**Table 4:** Texture profile analysis TPA of the two ice cream formulated with milk cream (traditional) and with the optimized emulsion, respectively.

Ice cream	Hardness (N)	Cohesiveness	Springiness	Adhesiveness (N × mm)	Chewiness (N)	Gumminess (N)
Traditional	1010.74^a^ (344.0)	3.865^a^ (0.6)	0.456^a^ (0.2)	− 157.225^a^ (28.8)	1497.7^a^ (847.7)	3539.75^a^ (931.2)
Optimized	1277.95^a^ (578.9)	6.515^b^ (0.9)	0.6925^a^ (0.3)	− 73.54^b^ (13.3)	3381.83^b^ (939.8)	6121.73^b^ (1691.3)

The values in parenthesis are the standard deviations.

^abc^In the same column, means without the same letter reveal significant differences (*p* < 0.05) according to the LSD multiple range test.

The viscosity of ice cream is an important parameter for determining the behavior of the fluid. Many factors contribute to ice cream viscosity, such as the interaction of hydrocolloids present in water, and other solutes (minerals)^45^. The flow curve of the ice cream made with HOPO was higher than that of the ice cream made without HOPO, which may be because the presence of pressurized and therefore denatured WP produced a more viscous ice cream. The apparent viscosity decreased as the shear rate increased, such that the ice creams displayed a pseudoplastic behavior.

## Conclusions

Through surface response methodology, this study verified the production of stable emulsions from whey without the need to work with isolated proteins (WPI), containing 4% of whey and 6.8% of oil concentration. However, the physicochemical composition of these whey can produce significant (P < 0.05) differences in variables such as ADS, PDI, ζ, *k, ƞ* and texture parameters, according to the results obtained. It is important to use standardization processes in the dairy industry to properly use WP residues and assess the costs and benefits of their use. Although previous studies have investigated the formation of oleogels through different methodologies (e.g., heat treatment, solvent exchange), in this investigation microfluidization was used because it is a high-pressure treatment that forms structured systems (more stable WP emulsions) and, thanks to the decreased ADS of WP aggregates, oleogels were formed without additional heat treatment. Because these gels are food-grade quality, they can be used in the food, cosmetic or pharmaceutical industries, generating added value by using a byproduct at low production costs. Finally, the concentration and type of oil used are significant factors in the manufacture of oleogels, as establishing the maximum amount of hydrophobic components in an emulsion will prevent the formation of crystals. It is possible to make an ice cream using an oleogel as a cream substitute without significant differences in viscosity and texture properties such as hardness and springiness. Therefore, oleogels are a promising fat substitute for ice cream production.
